# Prognostic analysis of gastric gastrointestinal stromal tumor with synchronous gastric cancer

**DOI:** 10.1186/1477-7819-12-25

**Published:** 2014-01-31

**Authors:** Mi Lin, Jian-Xian Lin, Chang-Ming Huang, Chao-Hui Zheng, Ping Li, Jian-Wei Xie, Jia-Bin Wang, Jun Lu

**Affiliations:** 1 Department of Gastric Surgery, Fujian Medical University Union Hospital, No.29 Xinquan Road, Fuzhou 350001, Fujian Province, China

**Keywords:** Gastrointestinal stromal tumor, Synchronous gastric cancer, Risk stratification, Prognosis

## Abstract

**Background:**

Many patients with gastric gastrointestinal stromal tumor (GIST) and synchronous gastric cancer have been described, most in single case studies. We retrospectively investigated the clinicopathologic features and prognostic effects of gastric GIST in patients with synchronous gastric cancer.

**Methods:**

The study enrolled 170 patients with gastric GIST, who had undergone complete surgical resection (R0) from January 2000 to December 2011. Forty-two patients had synchronous gastric cancer (CA Group), whereas 128 did not (Non-CA Group). The clinicopathologic features and potential prognostic factors in the two groups were compared.

**Results:**

Patients in the CA Group had more obvious symptoms, but a lower rate of preoperative diagnosis of gastric GIST (*P* <0.05). The two groups differed significantly in gender, age, greatest tumor diameter, risk stratification, tumor-associated ulcers, and CD117 and CD34 expression (*P* <0.05 each). Univariate analysis showed that age, risk stratification, postoperative oral imatinib and synchronous gastric cancer were predictive factors of survival (*P* <0.05). Cox regression analysis showed that risk stratification, postoperative oral imatinib and synchronous gastric cancer were independent predictors of survival (*P* <0.05). Stratified analysis showed that the 5-year overall survival rate was lower in patients with synchronous gastric cancer than in those without synchronous gastric cancer.

**Conclusions:**

Gastric GIST with synchronous gastric cancer had a lower rate of preoperative diagnosis, with correct diagnosis often missed. Survival, however, depended primarily on the gastric cancer.

## Background

Gastrointestinal stromal tumor (GIST) is the most common mesenchymal tumor of the gastrointestinal tract, with the most frequent site being the stomach. Since the first report of synchronous epithelial and stromal tumors in the stomach in 2000, [1] many patients with gastric GIST and synchronous gastric cancer have been described, most in single case studies [2-8]. However, little is known about the synchronous GIST and gastric cancer. Its clinicopathologic characteristics and prognostic factors are unclear. We therefore retrospectively compared clinicopathologic findings and prognostic factors in patients with primary GIST with those in patients with primary GIST and synchronous gastric cancer.

## Methods

Between January 2000 and December 2011, 194 patients diagnosed with primary gastric GIST underwent surgical treatment at the Affiliated Union Hospital of Fujian Medical University, Fuzhou, China. Patients were included if their diagnosis of GIST was confirmed pathologically after surgery and if they underwent initial complete surgical resection (R0) for GIST and/or gastric cancer at our hospital. Patients were excluded if they had malignancies other than gastric cancer along with gastric GIST; if they had distant metastases before surgery; or if their pathological diagnosis was incomplete. Of the 170 patients enrolled, 42 had synchronous gastric cancer (CA Group), and 128 did not (Non-CA Group).

Combinations of abdominal ultrasonography, computed tomography/magnetic resonance imaging, gastroscopy/endoscopic ultrasound were used for diagnosis of GIST/gastric cancer and for assessment of resectability. Metastatic disease was evaluated by computed tomography of the thorax, abdomen and pelvis and/or chest radiography. The surgical resection (enucleation, wedge resection, segmental resection and total/subtotal organ resection) of the GIST was performed according to the tumor site and size. All patients with gastric cancer underwent a D2 lymphadenectomy as described by the Japanese Classification of Gastric Carcinoma (JCGC) [9]. The risk stratification of GIST was according to the proposed modification of the NIH consensus classification for GIST [10]. The TNM stage of gastric cancer was based on the 7th edition of UICC/TNM system [11]. Patients classified as intermediate risk or high risk were suggested to receive 400 mg of imatinib orally after the operation, taken once daily with food, in the form of 100-mg capsules. The therapy was usually given for about 2 years for the intermediate risk and 3 years for the high risk.

The patients were followed up by trained investigators by mail, email, telephone, visits to patients or consultations at the outpatient clinic. The last follow-up date was February 2013. Survival duration was defined as the interval between the date of operation to the date of last contact, date of death, or date on which survival information was collected (due, for example, to loss of contact or death from other causes).

### Statistical analysis

All statistical analysis was performed using the Statistical Package for the Social Sciences (SPSS), version 18.0 for Windows (SPSS Inc, Chicago, USA). Measurement data were reported as means ± standard deviations, while enumerated data were assessed using the Chi-square or Fisher’s exact test. Kaplan-Meier curves were used to estimate overall survival time, with univariate comparisons between groups through the log-rank test. Multivariate analysis using the Cox model was used to evaluate independent predictors of survival. A *P* value <0.05 was considered statistically significant.

### Ethical approval

Ethics Committee of Fujian Medical University Union Hospital approved this retrospective study. Written consent was given by the patients for their information to be stored in the hospital database and used for research.

## Results

### Clinicopathologic features

In the 170 patients, there were 93 males and 77 females, with a male to female ratio of 1.21:1. The mean age at diagnosis of GIST was 61.1 ± 12.0 years. For GIST, 52 patients were classified as very low risk, 58 as low risk, 29 as intermediate risk, and 31 as high risk. In the CA Group, the staging of the synchronous gastric cancer was as follows: 14 patients were classified as Stage IA, 8 as Stage IB, 5 as Stage IIA, 1 as Stage IIB, 7 as Stage IIIA, 4 as Stage IIIB, and 3 as Stage IIIC. The histological subtype of the gastric cancer was as follows: 6 patients were classified as well differentiated, 21 as moderately differentiated, 10 as poorly differentiated and 5 as signet ring cell (SRC) histology. Compared with the Non-CA Group, the CA Group had a higher percentage of males, was older in age, and had a lower frequency of ulcer, a smaller greatest tumor diameter, lower risk stratification, and lower positivity rates for CD117 and CD34, with all of these differences being statistically significant (Table 1).

**Table 1:** Clinicopathologic features of all patients (cases (%))

**Items**	**Non-CA Group**	**CA Group**	P*
**Gender**			0.001*
Male/Female	61(47.7)/67(52.3)	32(76.2)/10(23.8)	
**Age(yr)**			0.001*
≤ 60/> 60	75(58.6)/53(41.4)	12(28.6)/30(71.4)	
**Tumor location**			
Upper	61(47.7)	14(33.3)	
Middle	45(35.2)	20(47.6)	
Lower	22(17.2)	8(19.0)	
**Greatest tumor diameter (cm)**			0.000*
≤ 2	19(14.8)	35(83.3)	
From >2 to 5	69(53.9)	7(16.7)	
From >5 to 10	24(18.8)	0(0.0)	
>10	16(12.5)	0(0.0)	
**Tumor bleeding**			
Yes/No	15(11.7)/113(88.3)	1(2.4)/41(97.6)	
**Tumor ulceration**			0.014*
Yes/No	36(28.6)/92(71.4)	4(9.5)/38(90.5)	
**Mitotic count**			
≤ 5/50 HPF	97(75.8)	38(90.5)	
From >5 to 10/50 HPF	21(16.4)	3(7.1)	
>10/50 HPF	10(7.8)	1(2.4)	
**Risk stratification**			0.000*
Very low	17(13.3)	35(83.3)	
Low	54(42.2)	4(9.5)	
Intermediate	27(21.1)	2(4.8)	
High	30(23.4)	1(2.4)	
**Tumor necrosis**			
Yes/No	7(5.5)/121(94.5)	1(2.4)/41(97.6)	
**Cystic tumor**			
Yes/No	10(.8)/118(92.2)	0(0.0)/42(100.0)	
**CD117**			0.009*
(−)/(+)	19(14.8)/109(85.2)	14(33.3)/28(66.7)	
**CD34**			0.000*
(−)/(+)	11(8.6)/117(91.4)	17(40.5)/25(59.5)	
**SMA**			
(−)/(+)	82(64.1)/46(35.9)	30(71.4)/12(28.6)	
**S-100**			
(−)/(+)	116(90.6)/12(9.4)	38(90.5)/4(9.5)	
**Postoperative complications**			
Yes/No	18(14.1)/110(85.9)	9(21.4)/33(78.6)	

*Analysis of variance. *P* <0.05 is significant. CA Group, gastric GIST patients with synchronous gastric cancer; Non-CA Group, gastric GIST patients without synchronous gastric cancer.

### Diagnosis

Of the 146 (85.9%) symptomatic patients, 97 had abdominal pain, 38 had abdominal tenderness, 33 had black stool, 32 had abdominal distension, 30 had weight loss, 18 had eructation, 16 had anorexia, 16 had sour regurgitation, 16 had hematemesis, 14 had an abdominal mass, 11 had a loss of strength, 11 had dysphagia, 7 had vomiting and 6 had nausea. The proportion of patients with symptoms was significantly higher in the CA than in the Non-CA Group (*P* <0.05) (Table 1). Of the 97 patients preoperatively diagnosed with gastric GIST, 50 were diagnosed by computed tomography, 38 by abdominal ultrasonography, 8 by magnetic resonance imaging, 2 by gastroscopy, and 37 by endoscopic ultrasound, while 8 patients were confirmed to have the disease by endoscopic biopsy pathology. GISTs in the remaining 73 patients were detected incidentally during surgery or by postoperative analysis of resected specimens, with patients being subsequently diagnosed with gastric GIST by postoperative pathology. Of the 128 patients in the Non-CA group, 88 tumors (68.8%) were identified before surgery but not confirmed by pathology, 8 (6.3%) were confirmed before surgery and 32 (25.0%) were confirmed after surgery. In the CA group, however, only 1 tumor (2.4%) was detected before surgery, whereas 41 (97.6%) were confirmed after surgery. The rate of preoperative diagnosis was significantly lower in the CA than in the Non-CA Group (2.4% versus 97.6%, *P* = 0.000) (Table 2).

**Table 2:** Diagnosis of all patients (cases (%))

**Items**	**Non-CA Group**	**CA Group**	P^*^
**Symptom**			0.012^*^
Symptomatic	105(82.0)	41(97.6)	
Asymptomatic	23(18.0)	1(2.4)	
**Diagnosis**			0.000^*^
Preoperative	96(75.0)	1(2.4)	
Postoperative	32(25.0)	41(97.6)	

*Analysis of variance. *P* <0.05 is significant. CA Group, gastric GIST patients with synchronous gastric cancer; Non-CA Group, gastric GIST patients without synchronous gastric cancer.

### Long-term surgical outcomes

**Figure 1:**
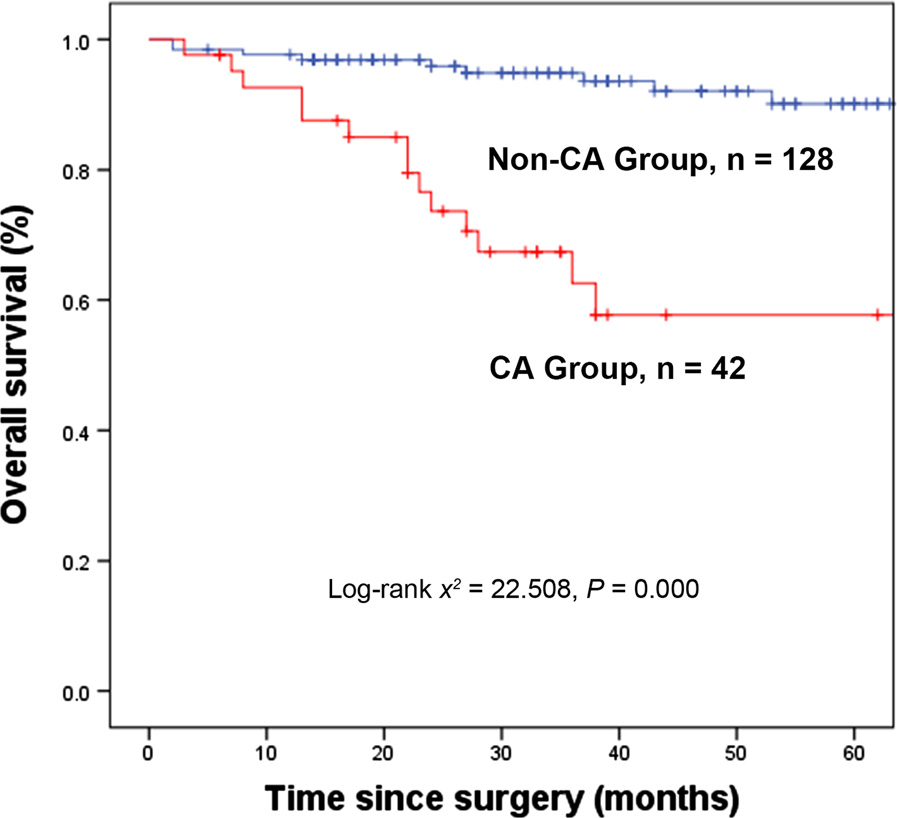
**Kaplan-Meier estimates of overall survival rates.** Kaplan-Meier estimates of overall survival rates relative to the presence or absence of synchronous gastric cancer in patients with gastrointestinal stromal tumor (GIST) (n = 170, χ2 = 22.508, *P* = 0.000). CA Group, gastric GIST patients with synchronous gastric cancer; Non-CA Group, gastric GIST patients without synchronous gastric cancer.

Of the 170 patients, 165 (97.1%) were followed up for 2 to 127 months (median, 38 months), including 40 patients (95.2%) in the CA Group and 125 (97.7%) in the Non-CA Group. During follow-up, 23 patients died, 14 in the CA and 9 in the Non-CA Group. The 3- and 5-year overall survival (OS) rates were 87.0% and 82.3%, respectively, for the entire cohort, 62.6% and 57.8%, respectively, for the CA group, and 94.8% and 90.1%, respectively, for the Non-CA group. The between-group differences were statistically significant (Figure 1).

### Univariate and multivariate survival analysis

Univariate analysis showed that patient age, risk stratification, postoperative oral imatinib and synchronous gastric cancer were predictive factors of survival (*P* <0.05; Table 3). Cox regression analysis showed that risk stratification, postoperative oral imatinib and synchronous gastric cancer were independent predictors of OS (*P* <0.05; Table 4).

**Table 3:** Univariate analysis of variables associated with survival in 170 patients with gastric gastrointestinal stromal tumor (GIST)

**Items**	**Cases (5-year survival rate,%)**	P*
**Gender**		
Male/Female	93(77.1)/77(88.2)	
**Age (yr)**		0.020*
≤ 60/>60	87(89.3)/83(74.2)	
**Tumor location**		
Upper/Middle/Lower	75(84.0)/65(76.3)/30(92.1)	
**Greatest tumor diameter (cm)**		
≤ 2/>2 to 5/>5 to 10/>10	54(77.3)/76(92.5)/24(78.4)/16(63.3)	
**Tumor bleeding**		
Yes/No	16(100.0)/154(80.1)	
**Tumor ulceration**		
Yes/No	40(92.3)/130(78.4)	
**Mitotic count (/50 HPF)**		
≤ 5/>5 to 10/>10	135(86.1)/24(67.9)/11(70.7)	
**Risk stratification**		0.004*
Very-low	52(76.2)	
Low	58(94.2)	
Intermediate	29(96.6)	
High	31(58.4)	
**CD117**		
(−)/(+)	33(64.8)/137(85.8)	
**CD34**		
(−)/(+)	28(71.2)/142(84.3)	
**SMA**		
(−)/(+)	112(82.2)/58(82.9)	
**S-100**		
(−)/(+)	154(82.3)/16(84.4)	
**Tumor necrosis**		
Yes/No	8(87.5)/162(81.7)	
**Cystic tumor**		
Yes/No	10(100.0)/160(81.6)	
**Synchronous gastric cancer**		0.000*
Yes/No	42(57.8)/128(90.1)	
**SRC in synchronous gastric cancer**		
Yes/No	5(40%)/37(59.2)	
**Postoperative complications**		
Yes/No	27(83.8)/141(82.2)	
**Postoperative oral imatinib**		0.009*
Yes/No	53(97.0)/117(77.3)	

*SRC*, signet ring cell.

*Analysis of variance. *P* <0.05 is significant.

**Table 4:** **Multivariate analysis of factors prognostic of survival in patients with gastric gastrointestinal stromal tumor** (**GIST) and synchronous gastric cancer**

**Parameters**	**β**	**SE**	**Wald**	P*	**RR**	**95% CI**
**Age**	−0.596	0.481	1.531		0.551	0.215-1.416
**Synchronous gastric cancer**	−2.296	0.602	14.571	0.000*	0.101	0.031-0.327
**Risk stratification**			24.190	0.000*		
**Very low versus high**	−2.504	0.607	17.001	0.000*	0.082	0.025-0.269
**Low versus high**	−2.544	0.682	13.895	0.000*	0.079	0.021-0.299
**Intermediate versus high**	−2.638	1.060	6.191	0.013*	0.071	0.009-0.571
**Postoperative oral imatinib**	2.213	1.045	4.489	0.034*	9.146	1.180-70.864

*Analysis of variance. *P* <0.05 is significant.

### Survival analysis based on risk stratification

**Figure 2:**
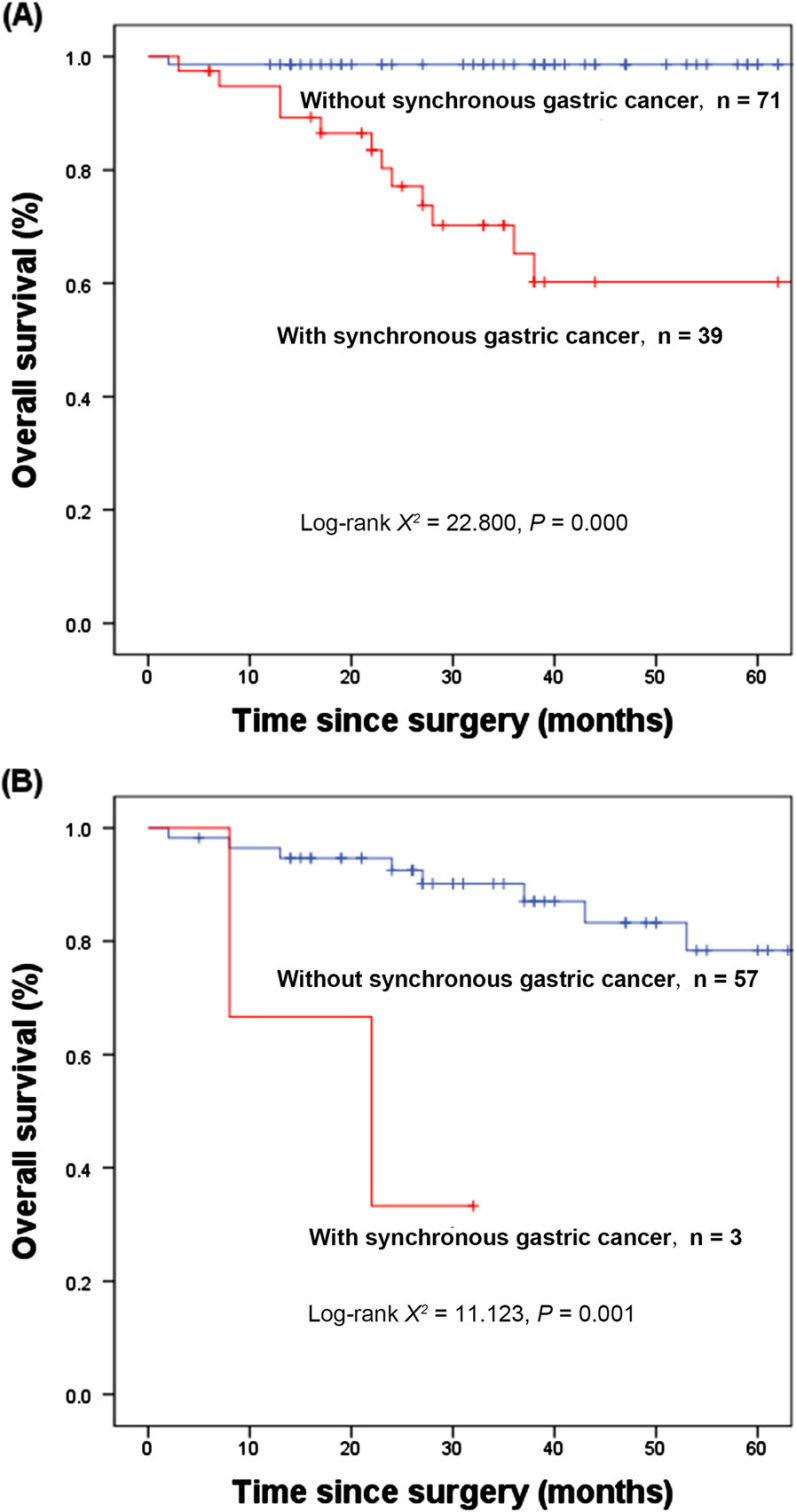
**Kaplan-Meier estimates of overall survival rates based on risk stratification.** Kaplan-Meier estimates of overall survival rates in gastric gastrointestinal stromal tumor (GIST) patients with and without synchronous gastric cancer and at **(A)** very low/low risk (n = 110, χ2 = 22.800, *P* = 0.000) and at **(B)** intermediate/high risk (n = 60, χ2 = 11.123, *P* = 0.001).

The 5-year survival rates were significantly lower among patients with synchronous gastric cancer than among patients without synchronous gastric cancer, both among patients stratified as being at very low risk/low risk (60.2% versus 98.6%, *P* <0.05) and among those stratified as being at intermediate risk/high risk (33.3% versus 98.1%, *P* <0.05) (Figure 2).

## Discussion

The incidence of GIST is only approximately 10 to 20 cases per million per year, [12-16] with gastric GIST being the most common type. Although GISTs are rare, the proportion of GIST patients who present synchronously with other malignancies is not low. In particular, the combination of gastric GIST and synchronous gastric cancer is relatively common. An analysis of 14 studies found that 4.5% to 33% of patients had GIST simultaneously with other neoplasms [17]. In our series we found that 42 of 170 (24.7%) patients with gastric GISTs presented with synchronous gastric cancers. Gastric GISTs accompanied by synchronous gastric cancer have specific pathological features. For example, 14 of 15 gastric GISTs with synchronous gastric cancer were smaller than 2.0 cm in size, with the fifteenth being 2.5 cm; moreover, almost all of these tumors were stratified as very low or low risk [2]. Similarly, we found that most of the gastric GISTs in patients with synchronous gastric cancer were small and of very low or low risk of malignancy. Moreover, only one of the 42 patients (2.4%) found to have gastric GIST with synchronous gastric cancer was diagnosed preoperatively, with all others detected incidentally during surgery or in postoperative pathology, a finding in agreement with previous results [1,2,18].

Clinical manifestations of gastric GIST were nonspecific, with some patients having no clinical manifestations when the tumor was small. The preoperative diagnosis of GIST depended mainly on imaging modalities, such as computed tomography and endoscopy [19,20]. In patients with simultaneous gastric cancer and gastric GIST, the symptoms of gastric GIST were often masked by the clinical symptoms of gastric cancer. Most of these patients had small GISTs (<2.0 cm) and saw a doctor for the symptoms of gastric cancer. Moreover, since most gastric GISTs were submucosal, muscular, or subserosal, patients often could not be preoperatively diagnosed by endoscopic biopsy. Furthermore, many clinicians lack the knowledge of multiple primary tumors and are satisfied with a diagnosis of gastric cancer alone, resulting in a low rate of preoperative diagnosis of gastric GIST.

Interestingly, we found that the gastric GIST patients with synchronous gastric cancer were older in age compared with those without synchronous gastric cancer, and that was a predictive factor of survival. However, the age was not an independent predictor of OS. We speculated that the finding might be associated with the high incidence of gastric cancer in older patients. In addition, the elderly might be with some change of gene expression profile and a lower immunity, resulting in more easily suffering from the synchronous tumors. Further studies are needed on the gene expression in primary tumor cells from older and younger patients and signal transduction may also provide us with some clues to this finding.

Common immunohistochemistry included CD117, CD34, SMA, S-100 of GIST were analyzed in our study, where we found statistically different positive rates of CD117 and CD34 between groups. However, further prognosis analysis suggested this finding was not related to prognosis. We found that the gastric GIST with synchronous gastric cancer had a lower positive rate of CD117 and CD34 based on the large sample. This was a finding not encountered before in the literature. It might be worth forming a base of classification of GIST tumors according to it. More research is needed.

Previously, GIST was associated with a poor prognosis, with 5-year OS rates after R0 resection ranging from 28% to 65%, [21-25] and another study reporting that patients with gastric GIST had a 5-year OS rate of 42% [26]. Additional studies, improvements in surgical skill, and the introduction of the molecular targeted drug imatinib have significantly improved the prognosis of patients with GIST, with a study in 2010 reporting a 5-year OS rate in 187 patients with gastric GIST being 75.9% [27]. Few studies to date have assessed the prognosis of patients with synchronous gastric GIST and gastric cancer. A study of 22 patients with gastric GIST and synchronous gastric cancer who underwent surgical treatment found that the 5-year OS rate was 57.8%, with a median survival time of 36 months [28]. We found that the 5-year OS rate in patients was significantly lower in gastric GIST patients with than without gastric cancer. Furthermore, risk stratification and the presence of synchronous gastric cancer were independent predictors of survival. The prognosis of gastric GIST patients was reported to be poorer for those with synchronous gastric cancer than for those without synchronous gastric cancer, regardless of risk stratification [4]. Similarly, we found that the 5-year OS rates were significantly lower in patients with synchronous gastric cancer than in those without synchronous gastric cancer, whether patients were stratified into the very low/low risk or intermediate/high risk groups. Thus, because of smaller tumor size, lower risk stratification and lower recurrence risk after complete resection, the prognosis of gastric GIST patients with synchronous gastric cancer was good, with GIST itself having little effect on patient prognosis. The main cause of poor prognosis in these patients was advanced synchronous gastric cancer, suggesting that active treatment of the synchronous gastric cancer would improve long-term survival of these patients.

Some studies revealed that SRC histology was associated with worse survival than non-SRC [29-31]. In our study, there were 5 patients with SRC carcinoma in the synchronous gastric cancer. To study the importance of SRC histology on survival, univariate analysis was done in the gastric GIST patients with SRC or with non-SRC, with a result of no significant differences. But the result might be of limitation of the small sample. Previous reports of the prognosis of patients with SRC were controversial. Some studies reported better 5-year survival rates in SRC than in other cell types in early gastric cancer [32,33]. However, others reported no significant differences when the stage of gastric cancer matched [34]. It has also been suggested that SRC histology is an independent predictor of poor prognosis in gastric cancer [29].

All GISTs are regarded as having malignant potential. Moreover, in patients with gastric GIST and synchronous gastric cancer, larger sized GISTs and higher risk stratification were associated with a high recurrence rate and poor prognosis, even after complete resection of the GIST [35]. Consequently, gastric GIST should be removed when incidentally discovered during surgery for gastric cancer; when necessary, targeted therapy should be considered.

## Conclusions

Gastric GIST with synchronous gastric cancer had a lower rate of preoperative diagnosis, with correct diagnosis often missed. Survival, however, depended primarily on the gastric cancer, suggesting that active treatment of the synchronous gastric cancer would improve long-term survival of these patients. Moreover, gastric GISTs should be removed when incidentally discovered during surgery for gastric cancer; when necessary, targeted therapy should be considered.

### Consent

Written informed consent was obtained from the patient for the publication of this report and any accompanying images.

## Abbreviations

GIST: gastrointestinal stromal tumor; OS: overall survival; SRC: signet ring cell.

## Competing interests

The authors declare that they have no competing interest.

## Authors’ contributions

ML, JXL and CMH conceived the study and participated in its design and coordination. ML, JXL, PL, JWX, JBW and JL helped to collect data. ML performed the statistical analysis. ML and JXL drafted the manuscript. CMH and CHZ helped revise the paper critically for important intellectual content. All authors read and approved the final manuscript.
